# Chest Binding and Care Seeking Among Transmasculine Adults: A Cross-Sectional Study

**DOI:** 10.1089/trgh.2018.0017

**Published:** 2018-12-14

**Authors:** Brooke A. Jarrett, Alexandra L. Corbet, Ivy H. Gardner, Jamie D. Weinand, Sarah M. Peitzmeier

**Affiliations:** ^1^ Department of Epidemiology, Johns Hopkins School of Public Health, Baltimore, Maryland.; ^2^ Department of Community Health Sciences, Boston University School of Public Health, Boston, Massachusetts.; ^3^ University of British Columbia School of Nursing, Vancouver, Canada.; ^4^ Boston University School of Medicine, Boston, Massachusetts.; ^5^ Department of Surgery, Oregon Health and Science University, Portland, Oregon.; ^6^ Department of Family Medicine, Memorial Medical Center, Las Cruces, New Mexico.; ^7^ Department of Health Behavior and Biological Sciences, Center for Sexuality and Health Disparities, University of Michigan School of Nursing, Ann Arbor, Michigan.

**Keywords:** breast, chest binding, health care access, help-seeking behaviors, transgender, transmasculine

## Abstract

**Purpose:** Chest binding, or compressing the chest tissue, is a common practice among transmasculine individuals that can promote mental health, but frequently results in negative physical health symptoms. The purpose of this study was to assess the prevalence and correlates of care seeking for binding-related health concerns among transmasculine adults.

**Methods:** Descriptive statistics were calculated and logistic regression models were run using data from the Binding Health Project, a cross-sectional online survey among transgender adults who had practiced chest binding (*n*=1800). The analysis was restricted to transmasculine individuals who had consistent access to health care and were female assigned at birth or intersex (*n*=1273).

**Results:** Of 1273 participants, 88.9% had experienced at least one binding-related symptom and 82.3% believed that it was important to discuss chest binding with their health care provider, while 14.8% had sought care related to binding. Participants reporting pain, musculoskeletal, or neurological symptoms had 3.19, 1.85, and 1.72 times the adjusted odds, respectively, of seeking care compared to those who did not report those symptoms (95% confidence intervals [CIs]: 1.38–7.37; 1.12–3.06; 1.10–2.68). Care seeking was associated with feeling safe and comfortable initiating a conversation about binding with one's provider (adjusted odds ratio [AOR]=2.07, 95% CI 1.32–3.24). Care seeking was not significantly associated with feeling comfortable receiving a chest examination (AOR=1.07, 95% CI 0.71–1.62).

**Conclusion:** Low rates of care seeking for binding-related symptoms may be driven by lack of access to a provider with whom patients feel safe and comfortable, rather than by general discomfort with chest examinations. While transmasculine patients may be most likely to present with musculoskeletal, neurological, or pain-related concerns, providers should also assess for other symptoms. Providers should be familiar with the benefits and potential complications of binding and initiate non-stigmatizing positive discussions about binding with their transmasculine patients.

## Introduction

Approximately 0.6% of the United States adult population identifies as transgender.^1^ Among transmasculine individuals (transgender individuals assigned a female sex at birth who identify on a spectrum of masculinity), breasts can cause significant gender dysphoria, that is, “discomfort or distress [due to] a mismatch between biological sex and gender identity.”^2,3^ As a result, transmasculine individuals may practice chest binding, a method of compressing the chest tissue to achieve a flatter chest contour.^4^ Common methods for binding include wearing one or multiple sports bras to flatten the chest; wrapping the chest with elastic bandages; and wearing commercial binders, which are undergarments that are specially designed, ultra-tight, and often made of nylon and spandex.^5^

While chest binding may have substantial mental health and safety benefits, the practice has also been previously associated with negative physical symptoms.^5,6^ Peitzmeier et al.^5^ found that 97.2% of individuals who bound their chests experienced at least one negative physical symptom from binding, the most common of which were back pain (53.8%), overheating (53.5%), chest pain (48.8%), and shortness of breath (46.6%). Potentially severe symptoms such as scarring (7.7%) and rib fractures (2.8%) were also reported.

Many factors can discourage transgender individuals from seeking care for concerning symptoms, both those related to chest binding and in general. Providers may discriminate against or provide poor-quality health care to transgender individuals.^7–9^ In the United States, one-third of transgender individuals have been either verbally harassed or refused treatment while seeking care, and nearly a quarter did not seek care because they feared being mistreated due to their gender identity.^10^ Decisions regarding care seeking are also impacted by whether a clinic is considered transgender-competent and whether a person has disclosed their identity to their health care provider.^2,11,12^ Given these barriers, transmasculine individuals may avoid or be unable to access needed medical care for binding-related health concerns.

Although negative physical symptoms are widely experienced by those who bind their chests, little is known about how often individuals seek care for these symptoms or what factors are associated with care seeking. The purpose of this study was to assess the prevalence and correlates of care seeking for binding-related health concerns among transmasculine individuals who were female assigned at birth or intersex, had practiced chest binding, and had consistent access to health care.

## Materials and Methods

### Study design and participants

For this cross-sectional study, we used data from the Binding Health Project, which recruited adults who had either current or previous experience with chest binding and were either intersex or female assigned at birth (*n*=1800). Data were collected between April 2 and May 31, 2014 with a 32-item online survey developed by a group that included individuals in medical school, public health graduate school, or who had previous experience binding their chests. The survey was also reviewed for terminology, sensitivity, and relevance by a four-member pilot group of purposively selected community members who identified as transmasculine and had experience with binding.

Study participants were recruited from social media networks; online transgender community forums; email lists serving lesbian, gay, bisexual, transgender, and queer (LGBTQ) communities; word-of-mouth referrals; and several LGBTQ centers with paper advertisements. Participants voluntarily accessed the survey through a web link, read an informed consent page, and indicated their consent by clicking a button to begin the survey.

For this analysis we focused on transmasculine individuals and excluded any survey respondent who identified as cisgender (*n*=31) or solely under a feminine identity without selecting any transgender, genderqueer, masculine, or other identity (*n*=16). The survey focused on potential intrapersonal (e.g., age and gender identity) and interpersonal factors (e.g., feeling safe and comfortable initiating a conversation with one's health care provider about binding) rather than institutional, community, or other structural factors that might influence care seeking.^13^

Although individuals without consistent access to health care are unlikely to seek care for binding-related symptoms simply because they are unable, individuals who do have consistent access to care will have other reasons for seeking or not seeking care. Therefore, we excluded any participant who self-reported no or inconsistent access to a health care provider. Access to care was assessed with two survey items that asked whether each participant's health care provider knew about their binding or had addressed their binding. Participants who selected “no, I do not have a health care provider,” “no, I do not have a consistent health care provider,” or “no, I do not have access to a health care provider” to either question (*n*=480) were excluded for a final sample size of 1273 participants. This research was declared exempt by the institutional review board of Boston University because the data were collected through an anonymous survey.

### Measures

Participants reported demographic characteristics, negative physical symptoms caused by binding, symptom severity, and health care engagement (Table 1). The outcome of interest was seeking care from a health care provider for a binding-related health concern. Participants were considered to have experienced the outcome if they reported that they had “experienced a health concern related to binding and sought care from a provider.” Participants were considered to not have experienced the outcome if they marked that they had not sought care from a provider, regardless of whether they reported having a health concern. We included those who reported not having a “health concern from binding” in our study population because 97% of participants in the full dataset reported at least one binding-related, negative physical symptom.^5^

**Table 1.** Definitions of Outcome and Covariates

Key variable	Type	Description
**Outcome**		
Sought care	Binary	Participants who responded, “Yes, I have experienced a health outcome from binding and sought care.”
**Demographics**		
Age	Continuous	—
Binds every day	Binary	Reported binding 7 days per week on average
Lifetime binding	Continuous	Total weeks of lifetime binding reported by the participant. Participants could select from 11 categories, each expressed as a range: 1–3 weeks, 1–3 months, 3–6 months, 6–9 months, 1 year, 2 years, 3 years, 4 years, 5 years, 6 years, and 7+ years. To be conservative, we took the lower end of the range and, if necessary, multiplied by 4 weeks per month, for example, a person who reported lifetime binding of 6–9 months was modeled as having bound for 24 weeks.
Sex	Binary	Self-reported either as female sex assigned at birth or intersex.
**Gender identities**		
Transgender, male or masculine, feminine, agender, genderqueer/bigender, masculine feminine, and a gender not listed above^a^	Binary	Participants could select from 25 predefined identities and write in an unlimited number of self-defined identities. Resulting identities were grouped into seven categories. The category “gender not listed” was not included in the multivariable model due to insufficient power.
**Negative symptom categories**		Reported experiencing at least one of the following symptoms as a result of binding:
Pain	Binary	Chest pain, shoulder pain, back pain, or abdominal pain
Musculoskeletal	Binary	Rib fractures, rib or spine changes, bad posture, shoulder joint “popping,” or muscle wasting
Neurological	Binary	Numbness, headache, light headedness, or dizziness
Gastrointestinal	Binary	Digestive issues or heartburn
General	Binary	Overheating, fatigue, or weakness
Respiratory	Binary	Cough, respiratory infections, or shortness of breath
Skin or Tissue	Binary	Chest tenderness, scarring, swelling, acne, itch, skin changes, chest changes, or skin infection
**Symptom severity**		
Severe pain	Binary	Pain rated 7 or greater on a scale of 1 (no pain) to 10 (worst pain) of the neck, chest, back, shoulder, or ribs/chest pain
Binding limits daily activities	Binary	—
Concern	Continuous	Scale from 1 (not concerned at all) to 5 (extremely concerned)
**Health care engagement**		
Thinks binding is important to discuss with their health care provider (HCP)	Binary	—
Feels safe and comfortable initiating a conversation with their HCP about binding	Binary	Response to “Do you feel safe/comfortable initiating a discussion about current or past binding with your primary care provider?” Participants could select multiple responses: “I feel safe,” “I feel comfortable,” “I do not feel safe,” “I do not feel comfortable,” or “no, I don't think it's important.” We affirmatively marked participants if they selected either “I feel safe” or “I feel comfortable” without selecting that they felt “unsafe” or “uncomfortable.”
Feels comfortable with their HCP conducting a chest examination	Binary	The survey indicated, “This question uses language about bodies that you may or may not use or identify with in respect to your own body. You may skip this question if you do not feel comfortable answering. The term “breast exam” is referencing a specific type of medical exam. Are you or would you feel comfortable with your health care provider conducting a breast exam?” We affirmatively marked participants if they selected, “Yes, I feel comfortable.”
Health care provider knows about binding practices	Binary	—
Manner in which health care provider addressed binding practice	Categorical	Participants could select, my health care provider addressed binding “positively/neutrally,” “negatively,” or “have never addressed it.”

^a^ Categories of gender identities were developed by an advisory focus group of transmasculine adults who had engaged in chest binding.

HCP, health care provider.

### Analyses

For covariates that we hypothesized to have an association with care seeking, we calculated simple descriptive statistics using means (standard deviation [SD]) for continuous variables, percentages for binary and categorical variables, and differences between groups who did and did not seek care using χ^2^ or *t*-tests as appropriate. We also calculated descriptive statistics for two variables hypothesized to result from the outcome: whether a provider was aware of the participant's binding practice and when presented with binding-related symptoms, whether the provider addressed the participant's practice negatively, positively or neutrally, or not at all.

We assessed all covariates, besides provider awareness and provider attitudes of binding, for associations with care seeking using bivariate logistic regression models. All covariates from the bivariate logistic regression models were included in a single multivariable logistic regression to determine adjusted associations with care seeking. Participants with missing data were excluded using model-wise deletion. Denominators for descriptive statistics and every bivariate model varied because we included any participant with data on that particular independent variable. For the multivariable model, we excluded participants with any missing data, which yielded a reduced, final sample size of *n*=1040. We conducted all analyses using STATA 14.1 at a significance level of <0.05.^14^

### Sensitivity analyses

Two covariates contained >5% missing data: comfort with a health care provider performing a chest examination (*n*=155 missing) and feeling safe and comfortable initiating a conversation with a health care provider about binding (*n*=87 missing). To assess the effect of using model-wise deletion even if the data were missing not at random, we conducted a sensitivity analysis by imputing participants with missing data as all answering in the negative (i.e., were not comfortable with a chest examination or did not feel safe initiating a conversation about binding) and then in the affirmative. Multivariable results using these assumptions did not differ substantively from the results obtained in the final multivariable model using model-wise deletion. A second sensitivity analysis was done by running the multivariable model with only participants who reported having a health concern (*n*=733). The associations were qualitatively similar to the results from the analysis using the full study population (*n*=1273).

## Results

### Descriptive analysis

Participants were a mean (SD) age of 25.6 (7.5) at the time of the survey and had bound their chest for a mean 135.9 (108.0) weeks in their lifetime (Table 2). In addition, 57.2% (*n*=728) reported binding their chest every day. A majority of participants identified as transgender (82.9%, *n*=1055) or masculine (70.7%, *n*=900). Few participants (14.8%, *n*=189) reported having sought care for a binding-related concern.

**Table 2.** Associations Between Care Seeking and Demographics, Negative Symptom Categories, Symptom Severity, and Engagement with Health Care

	*n*^a^	% Of total population	% Who sought care	*p*-Value^b^
**Outcome**				
Sought care				
Yes	189	14.8	100	—
No	1084	85.2	0	
**Demographics**				
Binds every day				
Yes	728	57.2	19.0	**0.00**
No	545	42.8	9.4	
Intersex				
Yes	16	1.3	18.8	0.720
No	1257	98.7	14.8	
Transgender				
Yes	1055	82.9	15.4	0.262
No	218	17.1	12.4	
Male or masculine				
Yes	900	70.7	15.9	0.104
No	373	29.3	12.3	
Genderqueer/bigender				
Yes	422	33.2	14.0	0.541
No	851	66.8	15.3	
Agender				
Yes	288	22.6	11.1	**0.043**
No	985	77.4	15.9	
Feminine				
Yes	116	9.1	7.8	**0.024**
No	1157	90.9	15.6	
Masculine feminine				
Yes	69	5.4	14.5	0.932
No	1204	94.6	14.9	
Gender not listed above				
Yes	6	0.5	0.0	0.600
No	1267	99.5	14.9	
**Negative symptom categories**				
Any symptom				
Yes	1132	88.9	16.3	**0.000**
No	141	11.1	2.8	
Pain category				
≥1 Symptom	952	74.8	18.5	**0.000**
No symptoms	321	25.2	4.0	
Musculoskeletal category				
≥1 Symptom	617	48.5	23.3	**0.000**
No symptoms	656	51.5	6.9	
Neurological category				
≥1 Symptom	530	41.6	22.6	**0.000**
No symptoms	743	58.4	9.3	
Gastrointestinal category				
≥1 Symptom	236	18.5	25.4	**0.000**
No symptoms	1037	81.5	12.4	
General category				
≥1 Symptom	813	63.9	18.6	**0.000**
No symptoms	460	36.1	8.3	
Respiratory category				
≥1 Symptom	658	51.7	19.2	**0.000**
No symptoms	615	48.3	10.2	
Skin/tissue category				
≥1 Symptom	989	77.7	17.0	**0.000**
No symptoms	284	22.3	7.4	
**Symptom severity**				
Reported pain ≥7 in neck, chest, back, shoulder, or ribs/chest pain				
Yes	498	39.1	24.9	**0.000**
No	775	60.9	8.4	
Binding limits daily activities				
Yes	268	21.0	29.5	**0.000**
No	1005	79.0	11.0	
**Engagement with health care**				
Thinks binding is important to discuss with their HCP				
Yes	1048	82.3	16.8	**0.000**
No	225	17.7	5.8	
Feels safe and comfortable initiating a conversation with their HCP about binding				
Yes	668	56.3	19.9	**0.000**
No	518	43.7	10.0	
Feels comfortable with their HCP conducting a chest examination				
Yes	523	46.8	15.1	0.226
No	595	53.2	12.6	

	Overall	Sought care	Did not seek care	*p*-Value
Mean age (SD)	25.6 (7.5)	27.3 (7.2)	25.3 (7.5)	**0.001**
*n*=1273		*n*=189	*n*=1084	
Mean lifetime	135.9 (108.0)	187.8 (108.3)	126.8 (105.4)	**0.000**
weeks of binding		*n*=189	*n*=1084	
*n*=1273				
Mean concern^c^	2.9 (1.3)	3.7 (1.1)	2.8 (1.2)	**0.000**
*n =* 1264		*n*=189	*n*=1075	

Boldface indicates statistical significance (*p*<0.05).

^a^ Total *n* varies by row, as only study participants with available data for each variable are presented.

^b^ *p*-Values for categorical values were calculated with a χ^2^ test or a two-sided Fisher's exact test if the expected cell count was ≤5. Comparisons for continuous variables were performed with *t*-tests based on variances and used Satterthwaite's method when variances were unequal.

^c^ Ranked on a scale from 1 (not concerned) to 5 (extremely concerned).

SD, standard deviation.

In total, 88.9% (*n*=1132) of participants had experienced at least one negative physical symptom. Participants most commonly reported symptoms from the skin and tissue (77.7%, *n*=989) and pain (74.8%, *n*=952) categories, while gastrointestinal (18.5%, *n*=236) and neurological (41.6%, *n*=530) were the least commonly cited categories. Severe pain caused by binding was reported by 39.1% (*n*=498) of participants, and 21.0% (*n*=268) reported that binding limited their daily activities.

A majority of participants (82.3%, *n*=1048) believed that binding was an important topic to discuss with their health care provider, and 56.3% (*n*=668) of participants felt safe and comfortable initiating that conversation. Fewer (46.8%, *n*=523) participants felt comfortable with their health care provider performing a chest examination. The mean concern among participants about the effect of binding on their health on a scale of 1 (not concerned at all) to 5 (extremely concerned) was 2.9.

Over half of participants (57.2%, *n*=727) reported that their provider was aware of their binding practice, and of those participants, 53.0% (*n*=385/727) had not discussed binding with their provider (Table 3). Of participants who had sought care for a binding-related concern from a health care provider who was aware of their binding practice (*n*=168), 29.2% (*n*=49) reported that their provider had never discussed binding with them, 17.3% (*n*=29) reported that their provider had addressed the practice negatively, and 53.6% (*n*=90) addressed the practice either positively or neutrally. Of participants who had never sought care for a binding-related concern but whose health care provider was aware of their binding practice (*n*=559), 60.1% (*n*=336) reported that their provider had never addressed the topic, 2.9% (*n*=16) had addressed the practice negatively, and 37.0% (*n*=207) addressed the practice either positively or neutrally.

**Table 3.** Health Care Provider Awareness of Chest Binding and Discussion of Chest Binding with Participant (*N*=1272)

	Overall % (n/N)	Among those who sought care % (n/N)	Among those who did not seek care % (n/N)
Health care provider was aware of participant's binding practice	57.2 (727/1272)	89.4 (168/188)	51.6 (559/1084)
Among participants whose provider was aware of their binding practice, how binding was addressed by the provider			
Was not addressed	53.0 (385/727)	29.2 (49/168)	60.1 (336/559)
Negatively	6.2 (45/727)	17.3 (29/168)	2.9 (16/559)
Positively or neutrally	40.8 (297/727)	53.6 (90/168)	37.0 (207/559)

One participant did not respond whether their health care provider was aware of the binding practice.

### Correlates of care seeking

No specific gender identity was independently associated with seeking care in the adjusted model, although participants who identified in the agender or feminine categories were significantly less likely to seek care in the bivariate analyses (Table 4). In the bivariate models, every symptom category was significantly associated with seeking care. In the multivariable model, participants reporting any kind of pain (adjusted odds ratio [AOR]=3.19, 95% confidence interval [CI]: 1.38–7.37), musculoskeletal symptoms (AOR=1.85, 95% CI: 1.12–3.06), or neurological symptoms (AOR=1.72, 95% CI: 1.10–2.68) were more likely to seek care than those who did not report those symptoms.

**Table 4.** Bivariate and Adjusted Associations Between Care-Seeking Behavior and Variables Related to Demographics, Negative Symptom Categories, Symptom Severity, and Engagement with Health Care

	Crude OR (95% CI)	*p*-Value^a^	Adjusted OR^b^ (95% CI)	*p*-Value
Demographics				
Age (each additional year)	1.03 (1.01–1.05)	**0.001**	1.02 (0.99–1.05)	0.150
Binds every day (vs. not)	2.26 (1.61–3.19)	**0.000**	1.31 (0.84–2.04)	0.232
Lifetime binding (per additional week)	1.00 (1.00–1.01)	**0.000**	1.00 (1.00–1.00)	**0.002**
Intersex (vs. female sex assigned at birth)	1.33 (0.38–4.71)	0.660	1.56 (0.26–9.15)	0.623
Identifies as transgender (vs. not)	1.28 (0.83–1.98)	0.263	0.90 (0.51–1.61)	0.734
Male or masculine	1.34 (0.94–1.92)	0.105	1.20 (0.74–1.92)	0.456
Genderqueer/bigender	0.90 (0.65–1.26)	0.541	1.30 (0.82–2.06)	0.258
Agender	0.66 (0.44–0.99)	**0.044**	0.93 (0.53–1.63)	0.801
Feminine	0.46 (0.23–0.92)	**0.028**	0.54 (0.24–1.21)	0.134
Masculine feminine	0.97 (0.49–1.93)	0.932	1.23 (0.51–2.95)	0.642
Negative symptom categories				
Pain (yes vs. no)	5.37 (3.01–9.58)	**0.000**	3.19 (1.38–7.37)	**0.007**
Musculoskeletal	4.13 (2.90–5.90)	**0.000**	1.85 (1.12–3.06)	**0.017**
Neurological	2.86 (2.07–3.94)	**0.000**	1.72 (1.10–2.68)	**0.018**
Gastrointestinal	2.40 (1.70–3.39)	**0.000**	1.09 (0.69–1.73)	0.702
General symptoms	2.53 (1.74–3.69)	**0.000**	1.00 (0.60–1.66)	0.993
Respiratory	2.08 (1.50–2.87)	**0.000**	1.06 (0.68–1.65)	0.810
Skin/tissue	2.56 (1.59–4.12)	**0.000**	1.10 (0.60–2.04)	0.748
Symptom severity				
Reported severe pain (yes vs. no)	3.62 (2.62–5.01)	**0.000**	1.15 (0.74–1.81)	0.533
Binding limits daily activities	3.40 (2.45–4.72)	**0.000**	1.84 (1.18–2.87)	**0.007**
Concern 1=not at all concerned 5=extremely concerned	1.88 (1.64–2.16)	**0.000**	1.37 (1.14–1.66)	**0.001**
Engagement with health care				
Thinks binding is important to discuss with their HCP (vs. not)	3.29 (1.84–5.90)	**0.000**	1.23 (0.54–2.80)	0.624
Feels safe and comfortable initiating a conversation with their HCP about binding	2.23 (1.58–3.14)	**0.000**	2.07 (1.32–3.24)	**0.001**
Feels comfortable with their HCP conducting a chest examination	1.23 (0.88–1.73)	0.227	1.07 (0.71–1.62)	0.754

Bolded *p*-values indicate significance at *p*<0.05.

^a^ Unadjusted *p*-values calculated using Wald test, and adjusted *p*-values calculated using likelihood ratio tests.

^b^ The multivariable model includes all 23 covariates and excludes any participant with missing data for a final, reduced sample size of 1040 of 1273 possible participants.

OR, odds ratio; CI, confidence interval.

In terms of symptom severity, reporting severe pain was statistically associated with care seeking in the bivariate model (odds ratio=3.62, 95% CI: 2.62–5.01) but was not significant in the multivariable model. Participants who reported higher levels of concern about the effect of binding on their health were more likely to have sought care (AOR=1.37, 95% CI: 1.14–1.66). With respect to engagement in the health care system, those who felt safe and comfortable initiating a conversation with their health care provider were significantly more likely to have sought care than those who did not feel safe and comfortable initiating such a discussion (AOR=2.07, 95% CI: 1.32–3.24). Feeling comfortable receiving a chest examination was not statistically associated with care seeking in either model.

## Discussion

Although most participants had experienced at least one negative physical symptom and many reported severe pain or found that binding limited their daily activity, only 14.8% sought care for their binding-related health concerns. Despite relatively low care-seeking behavior, 82.3% of participants believed that it was important to have a conversation about chest binding with their health care provider, suggesting that there are additional barriers to seeking care.

One of the strongest predictors of care seeking in both the bivariate and multivariable models was whether the individual felt safe and comfortable initiating a conversation about binding with their health care provider. This is consistent with extensive literature demonstrating that stigma and discrimination in health care settings are considerable barriers for transgender patients.^10–12,15–19^ This finding is particularly striking since both the bivariate and multivariable models demonstrated that feeling comfortable with one's health care provider performing a chest examination was not significantly associated with care seeking. This supports other literature that shows that while some transgender patients may dislike undergoing physical examinations such as Pap tests due to discomfort and gender dysphoria, a trusting relationship with one's provider is able to increase patient willingness to engage in care.^20,21^ In addition to our findings around facilitating chest examinations to assess and treat symptoms in transmasculine patients, more work is needed about chest examinations for preventive health services, such as mammograms, in this patient population.^22^

Previous research suggests that provider empathy, knowledge about gender-affirming care, and willingness to open-mindedly discuss transgender-specific care may help cultivate better relationships between providers and their transgender patients.^23^ This study demonstrates, however, that nearly one-third of participants who sought care for a binding-related health concern reported that their provider avoided the topic of binding altogether despite knowing about the participant's practice. Furthermore, among participants who had sought care from a provider who knew about their practice, more than one in six reported that their provider addressed their binding negatively. Given the central importance of chest binding for identity affirmation, safety, and mental health in the daily lives of many transmasculine individuals, these findings underscore the continued need for clinicians to be familiar with the standards of care for transgender patients and to practice gender-affirming, non-stigmatizing clinical care for transgender individuals.^2,5,6,24,25^

We believe that patient and provider education about binding-related symptoms can begin immediately, especially as there are clear medical interventions for certain binding-related outcomes like acne and rib fractures. While health care providers may not see a clear intervention for other symptoms such as pain or tissue changes, they should still initiate a conversation with their patients. Conversations should focus on how binding-related symptoms may impact other aspects of the patient's health, for example, how skin and soft tissue changes can compromise top surgery outcomes,^26–28^ and to help the patient think through benefits, risks, and risk mitigation tactics. Although evidence-based clinical guidelines for minimizing binding-related risks do not exist, a previous analysis of these same cross-sectional data demonstrated that taking days off from binding and specific methods of binding were associated with a reduced risk of negative health outcomes.^5^ More research, especially prospective data on mitigating binding-related risks and how binding may impact top surgery outcomes, is needed for robust, evidence-based clinical recommendations.

In their conversations about binding-related health concerns, providers should also be aware that not all symptoms motivate individuals to seek care. Musculoskeletal, neurological, and pain-related symptoms were significantly associated with care seeking in both bivariate and multivariable models, while respiratory, gastrointestinal, skin, tissue, and general symptoms were only significant in the bivariate models. While patients may be most likely to present with musculoskeletal, neurological, or pain-related concerns about binding, providers should also assess those patients for other symptoms. Clinicians should also consider, given the high levels of binding-related symptoms, initiating a discussion about the practice of binding with any patient who binds their chest, regardless of whether they present with binding-related health concerns.

Finally, the presence of severe pain was associated with care seeking in the bivariate model but not in the multivariable model, which also controlled for any pain. This suggests that while pain is an important predictor of whether individuals will seek care, those who experience severe pain are no more likely to seek care than those who experience less severe pain. Furthermore, after controlling for pain severity, those who expressed greater concern about the effects of chest binding on their health were more likely to seek care. This suggests that even among those with high symptom severity, some individuals may minimize the effects of chest binding on their health, resulting in artificially low levels of concern and thus of care seeking. Future research is needed on whether warranted care seeking could be promoted with interventions that sensitize transgender individuals to feel appropriate concern for physical health risks related to chest binding.

### Limitations

There were several limitations to this study. Due to the limited scope of the analysis, the results are only generalizable to transmasculine patients with access to health care. Many transmasculine individuals do not seek care for binding-related symptoms because they cannot seek care for any medical issue as a result of discrimination in health care settings or lack of insurance.^10–12,15–19^ The unique barriers and facilitators to care seeking in this segment of the population are worthy of further study. However, these institutional and structural factors were not captured in this survey. Some intra- and interpersonal factors often included in studies about care seeking, such as income, education, and symptom duration, were also not included to distribute a survey of acceptable length that would be applicable to potential study participants from many international settings with different currencies, culturally defined racial categories, and education systems.

The survey also only collected cross-sectional information about binding, hence excluding time-varying aspects of binding such as changes in binding practices (e.g., reducing the number of hours per day spent binding in response to negative symptoms), which may have contributed to whether a participant sought care. Similarly, we did not fully assess reasons why participants did not discuss binding with their providers, including such factors as the belief that there was no medical treatment for the symptoms being experienced. Furthermore, the survey did not distinguish between current and previous binding, which may have influenced care seeking and precluded us from assessing secular trends.

Our analysis was also largely limited to the United States, United Kingdom, and Canada and was thus underpowered to study the effect of geography, culture, and health care systems on care-seeking behavior. There may have also been selection biases during recruitment, as those who self-selected to participate in the survey could have been more likely to have experienced health issues related to binding. Despite these limitations, these findings represent the first data on care seeking for binding-related symptoms among transmasculine individuals using a robust sample of over 1000 individuals and add to the growing literature on factors associated with transgender-specific access to care.

## Conclusion

Despite the high prevalence of negative physical symptoms in transgender individuals who bind their chests, care seeking for binding-related health concerns is relatively rare, even among those experiencing severe pain or limitations on daily activities. This analysis demonstrated that care-seeking behavior is associated with health care providers who create a safe, comfortable, and trans-friendly care environment. Providers should be familiar with the practice of binding, aware of its complications, and initiate non-stigmatizing positive discussions about binding with their transmasculine patients.

## Abbreviations Used

AOR: adjusted odds ratio
CI: confidence interval
HCP: health care provider
SD: standard deviation
